# Supplementary material for: An annual cycle of gene regulation in the red-legged salamander mental gland: from hypertrophy to expression of rapidly evolving pheromones
Source: BMC Dev Biol. 2019 Apr 27;19:10. doi: 10.1186/s12861-019-0190-z (PMC6487043; doi:10.1186/s12861-019-0190-z)
Supplement: Supplementary file 1 — Figure S1. P. shermani mental gland cDNA. Figure S2. qRT-PCR analysis of select mental gland genes. Figure S3. CIRBP affinity for different RNAs. Figure S4. EMSA with CIRBP domains. Figure S5. CIRBP – RNA interactions stabilized by formaldehyde crosslinking. (PDF 1358 kb) [file 12861_2019_190_MOESM1_ESM.pdf]

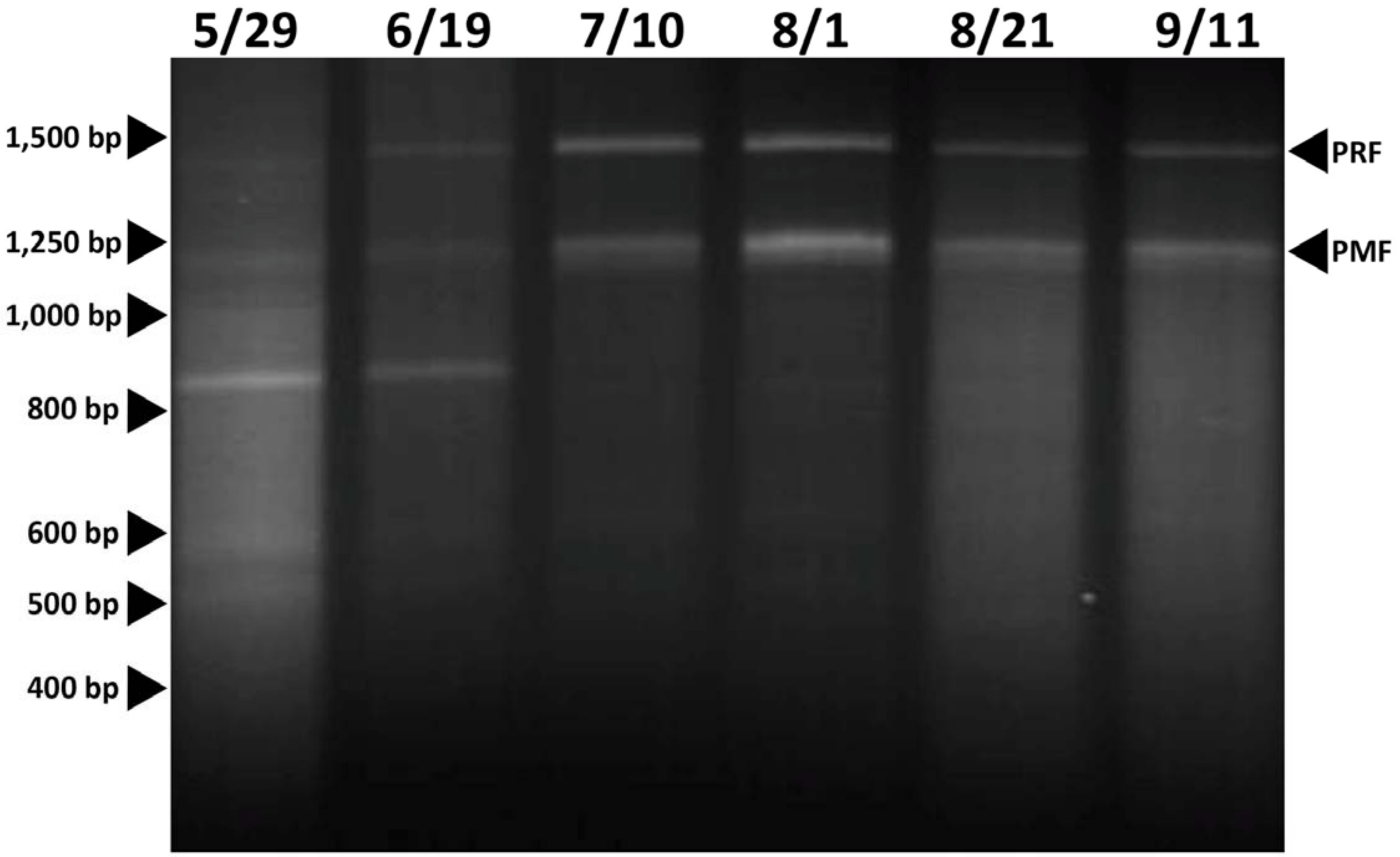


**Figure S1.** ***P. shermani*** **mental gland cDNA.**

Comparison of pooled, PCR amplified cDNA from six time points in mental gland development used for transcriptome sequencing. Arrows denote the bands corresponding to PRF and PMF mRNA, which are present at all 6 points.

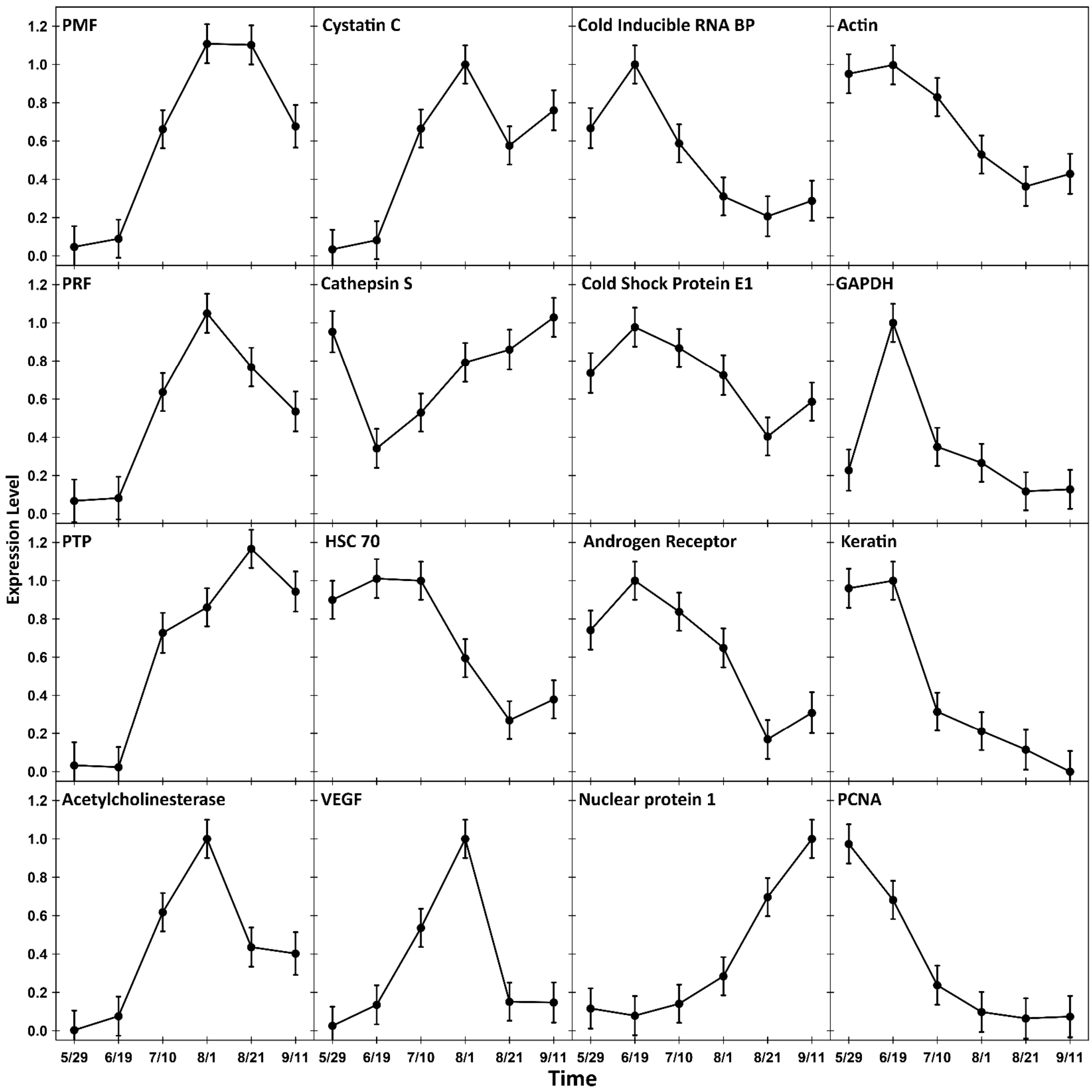


**Figure S2. qRT-PCR analysis of select mental gland genes.**

Transcript abundance was measured for 16 mental gland genes qRT-PCR, scaled relative the time point with the most abundant expression, and fit to a linear mixed effect model with gene and time as fixed effects and male as a random effect. Reported values are model estimated mean ± standard error.

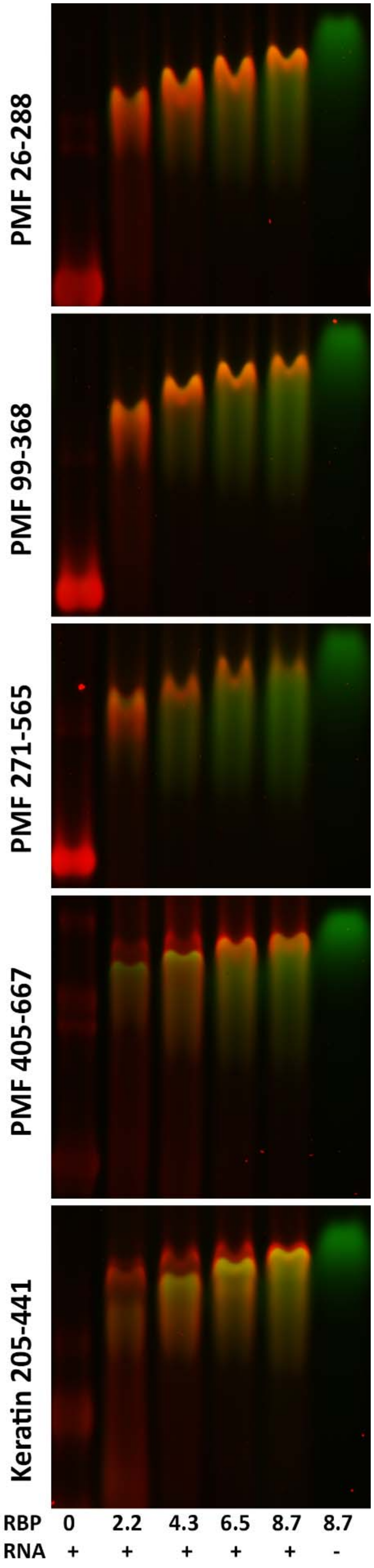


**Figure S3. CIRBP affinity for different RNAs.**

Overlays of fluorescent EMSAs using increasing concentrations rCIRBP/ECFP (RBP) with 200 ng of five different RNA molecules (four overlapping ~250 bp segments of the PMF 3’ UTR, and a Keratin 3’ UTR control). Protein fluorescence was detected by ECFP (green), and RNA was stained using Sybr Green II (red).

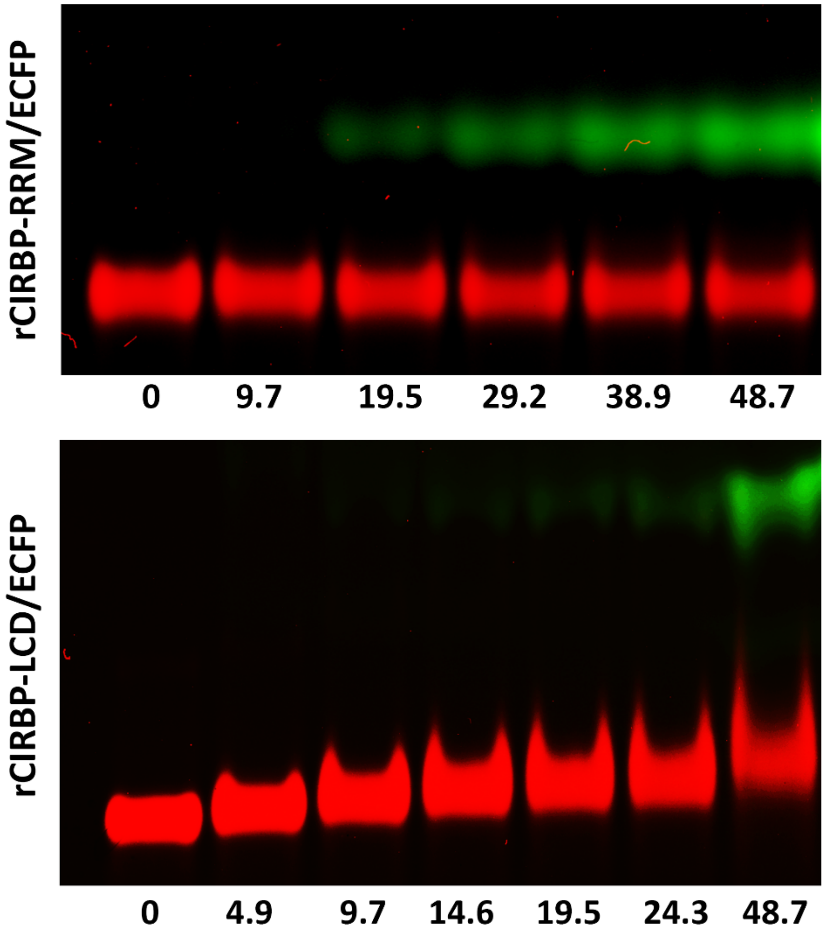


**Figure S4. EMSA with CIRBP domains.**

TAMRA-labeled PMF 3' UTR 99-368 RNA (30 ng; red) with increasing concentrations of either rCIRBP-RRM/ECFP or rCIRBP-LCD/ECFP (µM; green).

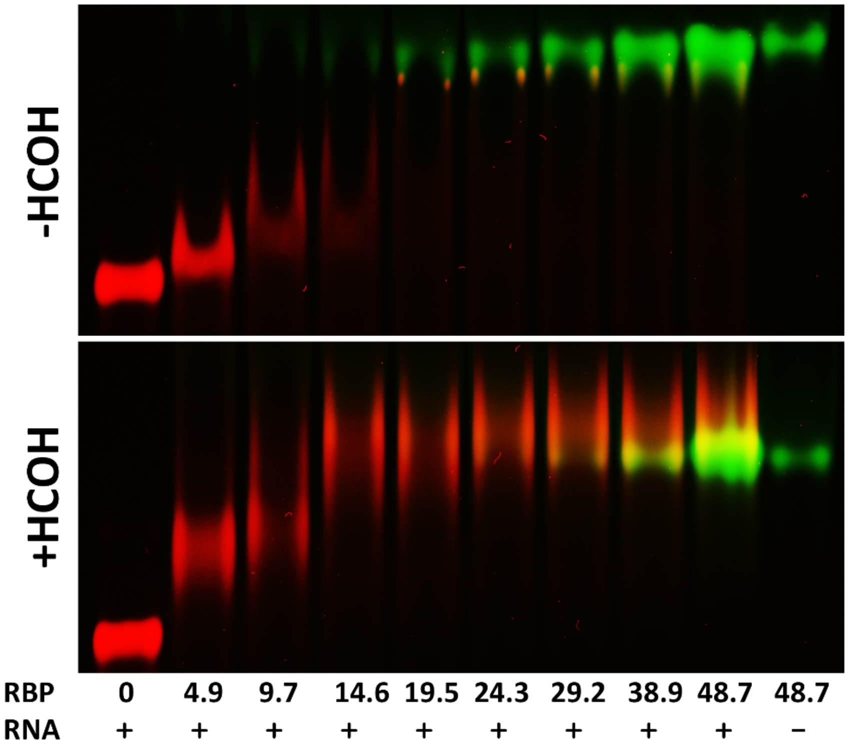


**Figure S5. CIRBP – RNA interactions stabilized by formaldehyde crosslinking.**

EMSA between TAMRA-labeled PMF 3' UTR 99-368 RNA (30 ng; red) and increasing concentrations of rCIRBP/ECFP (µM; RBP; green), with and without pre-treatment with 1% formaldehyde.
