# Supplementary material for: An annual cycle of gene regulation in the red-legged salamander mental gland: from hypertrophy to expression of rapidly evolving pheromones
Source: BMC Dev Biol. 2019 Apr 27;19:10. doi: 10.1186/s12861-019-0190-z (PMC6487043; doi:10.1186/s12861-019-0190-z)
Supplement: Supplementary file 2 — Table S1. Summary of P. shermani mental gland transcriptome. Table S2. Select list of differentially expressed genes. Table S3. MANOVA results for CIRBP expression and regulation. Table S4. Primers used for qRT-PCR, CIRBP expression, and in vitro transcription. (PDF 168 kb) [file 12861_2019_190_MOESM2_ESM.pdf]

Table S1. Summary of *P. shermani* mental gland transcriptome.

Total no. of reads	146,829,960
Average read length	100 bp
Total number of Trinity components	56,083
Total number of Trinity transcripts	158,361
Mean length of transcripts	578 bp
Median length of transcripts	394 bp
No. of Trinotate-predicted ORFs	47,097
Trinotate ORFs with SwissProt match (blastp)	22,182
Trinotate ORFs with TrEMBL match (blastp)	25,184
Transcripts with TrEMBL match (blastx)	39,600
Successfully re-aligned forward reads	66,392,752 (90.4%)

Table S2. Select list of differentially expressed genes. Comparison denotes the two time points compared by EBSeq, with fold change as the ratio of the second time point to the first, and the posterior probability of differential expression (PPDE).

Gene	Putative function	Comparison	Fold Change	PPDE
PMF Class II	Pheromone	6/19 → 8/1	28 X	1
C3	Putative pheromone	6/19 → 8/1	430 X	1
Sodefrin-like factor	Putative pheromone	6/19 → 8/1	350 X	0.962
VIP-like homolog	Putative pheromone	6/19 → 8/1	170 X	1
Calmodulin	Ca^{2+} binding	6/19 → 8/1	0.24 X	1
Parvalbumin	Ca^{2+} binding	5/29 → 6/19	9200 X	0.999
		6/19 → 8/1	0.00031 X	0.973
Calreticulin	Ca^{2+} binding	6/19 → 8/1	3.1 X	1
Kazal-type serine protease inhibitor	Secreted protease inhibitor	6/19 → 8/1	280 X	0.976
Cystatin	Secreted protease inhibitor	6/19 → 8/1	30 X	1
Cathepsin S	Cysteine protease	6/19 → 8/1	1.5 X	0.999
Protein disulfide isomerase A6	Chaperone	6/19 → 8/1	9 X	1
ADP-ribosylation factor 4	Vesicle transport	6/19 → 8/1	1.7 X	0.999
Gap junction protein	Gap junctions	6/19 → 7/10	480 X	0.999
		8/1 → 8/21	0.0012 X	1
Vascular endothelial growth factor A	Promotes angiogenesis	6/19 → 8/1	39 X	0.957
Tubulin α1	Microtubule formation	6/19 → 8/1	0.41 X	1
Histone H2A	Chromatin binding	6/19 → 8/1	0.36 X	0.999
Histone H1E	Chromatin binding	5/29 → 6/19	0.0074 X	0.935
eIF4AI	Translation initiation	5/29 → 6/19	31 X	0.909
		8/1 → 8/21	0.045 X	0.990
eIF 2	Translation initiation	5/29 → 6/19	0.011 X	0.901
Ribosomal protein L36	Translation	6/19 → 7/1	20 X	0.881
		8/1 → 8/21	55 X	0.997
Ribosomal protein L38	Translation	8/1 → 8/21	0.065 X	0.999
Ribosomal protein S4	Translation	8/1 → 8/21	0.049 X	0.984
Ribosomal protein S25	Translation	8/1 → 8/21	0.071 X	0.978

Table S3. MANOVA results for CIRBP expression and regulation. Statistically significant coefficients ($p < 0.05$) are bolded.

	Variable	Sum of Squares	F-statistic	p-value	Coefficient
R_{CIRBP} ~ *time*					
	time	**5.42×10^{-16}**	**23.18**	**5.0×10^{-5}**	**8.65×10^{-9}**
	Residuals	6.31×10^{-16}			
R_{Cath} ~ *time*					
	time	**2.55×10^{-16}**	**57.83**	**3.52×10^{-8}**	**5.93×10^{-9}**
	Residuals	1.19×10^{-16}			
$P_{Total\text{-}CIRBP}$ ~ *time x* R_{CIRBP}					
	time	**9.59×10^{8}**	**90.46**	**8.73×10^{-10}**	**-6.34×10^{3}**
	**R_{CIRBP}**	**1.65×10^{8}**	**15.60**	**5.63×10^{-4}**	**1.45×10^{12}**
	***time* : R_{CIRBP}**	**2.01×10^{8}**	**18.97**	**1.98×10^{-4}**	**-1.28×10^{12}**
	Residuals	2.65×10^{8}			
$P_{Intact\text{-}CIRBP}$ ~ *time x* R_{CIRBP} *x* $P_{Total\text{-}CIRBP}$ *x* R_{Cath}					
	time	**2.68×10^{7}**	**6.02**	**0.0290**	**-2.66×10^{3}**
	R_{CIRBP}	1.04×10^{7}	2.34	0.150	4.27×10^{12}
	**$P_{Total\text{-}CIRBP}$**	**4.91×10^{7}**	**11.02**	**0.00553**	**-0.382**
	R_{Cath}	1.80×10^{6}	0.41	0.536	-4.75×10^{14}
	time : R_{CIRBP}	2.00×10^{6}	0.45	0.514	-4.81×10^{12}
	time : $P_{Total\text{-}CIRBP}$	1.12×10^{6}	0.25	0.624	-1.60×10^{-2}
	***time* : R_{Cath}**	**2.95×10^{7}**	**6.63**	**0.023**	**4.74×10^{14}**
	R_{CIRBP} : $P_{Total\text{-}CIRBP}$	6.06×10^{5}	0.14	0.718	-1.91×10^{8}
	R_{CIRBP} : R_{Cath}	1.43×10^{7}	0.32	0.581	3.47×10^{22}
	$P_{Total\text{-}CIRBP}$: R_{Cath}	2.48×10^{7}	0.56	0.469	2.12×10^{10}
	time : R_{CIRBP} : $P_{Total\text{-}CIRBP}$	1.29×10^{7}	0.29	0.600	2.25×10^{8}
	time : R_{CIRBP} : R_{Cath}	1.96×10^{6}	0.04	0.837	-3.46×10^{22}
	time : $P_{Total\text{-}CIRBP}$: R_{Cath}	2.95×10^{7}	0.66	0.430	-2.10×10^{10}
	R_{CIRBP} : $P_{Total\text{-}CIRBP}$: R_{Cath}	7.92×10^{6}	0.18	0.680	-1.43×10^{18}
	***time* : R_{CIRBP} : $P_{Total\text{-}CIRBP}$: R_{Cath}**	**2.86×10^{8}**	**6.42**	**0.025**	**1.42×10^{18}**
	Residuals	5.79×10^{8}			
R_{PMF} ~ *time x* $P_{Total\text{-}CIRBP}$ *x* $P_{Intact\text{-}CIRBP}$					
	time	**1.50×10^{-10}**	**43.98**	**1.45×10^{-6}**	**4.97×10^{-6}**
	$P_{Total\text{-}CIRBP}$	2.40×10^{-13}	0.07	0.792	6.00×10^{-12}
	$P_{Intact\text{-}CIRBP}$	1.00×10^{-13}	0.004	0.950	8.49×10^{-12}
	time : $P_{Total\text{-}CIRBP}$	2.46×10^{-12}	0.72	0.406	1.05×10^{-9}
	time : $P_{Intact\text{-}CIRBP}$	6.00×10^{-14}	0.02	0.899	-1.03×10^{-10}
	$P_{Total\text{-}CIRBP}$: $P_{Intact\text{-}CIRBP}$	3.00×10^{-14}	0.01	0.922	-5.80×10^{-16}
	time : $P_{Total\text{-}CIRBP}$: $P_{Intact\text{-}CIRBP}$	7.50×10^{-13}	0.22	0.644	8.22×10^{-14}
	Residuals	7.18×10^{-11}			
P_{PMF} ~ *time x* R_{PMF} *x* $P_{Intact\text{-}CIRBP}$ *x* $P_{Total\text{-}CIRBP}$					
	time	**2.8585**	**128.28**	**4.17×10^{-8}**	**1.51**
	R_{PMF}	0.0451	2.02	0.178	-2.67×10^{-7}
	$P_{Intact\text{-}CIRBP}$	0.0373	1.67	0.218	-2.73×10^{-18}
	$P_{Total\text{-}CIRBP}$	0.0035	0.16	0.698	-3.23×10^{-19}
	time : R_{PMF}	0.0013	0.06	0.810	-1.39×10^{5}
	***time*: $P_{Intact\text{-}CIRBP}$**	**0.3478**	**15.61**	**0.00166**	**-4.49×10^{-4}**
	time : $P_{Total\text{-}CIRBP}$	0.0281	1.26	0.282	-1.48×10^{-4}
	R_{PMF} : $P_{Intact\text{-}CIRBP}$	0.0001	0.004	0.951	1.47×10^{-10}
	R_{PMF} : $P_{Total\text{-}CIRBP}$	0.0234	1.05	0.324	1.05×10^{-11}
	$P_{Intact\text{-}CIRBP}$: $P_{Total\text{-}CIRBP}$	0.0012	0.06	0.818	1.06×10^{-22}
	time : R_{PMF} : $P_{Intact\text{-}CIRBP}$	0.0003	0.01	0.916	18.1
	time : R_{PMF} : $P_{Total\text{-}CIRBP}$	0.0000	0.001	0.980	32.8
	time : $P_{Intact\text{-}CIRBP}$: $P_{Total\text{-}CIRBP}$	0.0295	1.33	0.270	5.63×10^{-8}
	R_{PMF} : $P_{Intact\text{-}CIRBP}$: $P_{Total\text{-}CIRBP}$	0.0060	0.27	0.614	-5.76×10^{-15}
	time : R_{PMF} : $P_{Intact\text{-}CIRBP}$: $P_{Total\text{-}CIRBP}$	0.0002	0.01	0.930	-8.35×10^{-3}
	Residuals	0.2897			

Table S4. Primers used for qRT-PCR, CIRBP expression, and *in vitro* transcription.

Primer	Sequence
**qPCR primers**	
PMF Class I 3'UTR 26-45 F'	TGA GGA ATC ACA CTG GCA TC
PMF Class I 3'UTR 103-82 R'	GCT CCT TGT TTT CTC TGG TAG C
PRF 3'UTR 97-116 F'	ATC GTG GGA TGG ACA GTT TG
PRF 3'UTR 186-167 R'	TCG GGA AAG ATG AGG ACT TC
PTP CDS 79-98 F'	CAC CTG CAG TCA GCT TTT TG
PTP CDS 164-145 R'	ATC CAG CCA TCA TCA TCC TC
CIRBP CDS 44-63 F'	TTG ACA CAA ACG AGC AGG AC
CIRBP CDS 187-168 R'	CGT CTT TCG CAT CTT CTT GG
Actin CDS 134-153 F'	TTG GTA TGG GCC AGA AAG AC
Actin CDS 241-222 R'	CAT CCC AGT TGG TGA CAA TG
Cathepsin S CDS 220-239 F'	GCA GCA AAG CTT GAT CTT CC
Cathepsin S CDS 359-340 R’	ACA CGG TCT GAA ATG GCT TC
Cystatin C CDS 134-153 F'	TTG CCA TGA CCG AGT ACA AC
Cystatin C CDS 275-256 R'	GGG TGT GTG CAA GTT GTT TG
PCNA 200-219 F'	CTG TGG GGG TTA AAA TGA GC
PCNA 296-277 R'	ATG GTG TCG GCA TTG TCT TC
Androgen Receptor 2-21 F'	TGG AAG CCA TTG AGC CTA TC
Androgen Receptor 147-128 R'	AAA ACC TGG TAG GGC TTT CG
Keratin 488-507 F'	TCG ATA AGG TCC GAT TCC TG
Keratin 603-584 R’	TAA GGG CTC CAT GTT GTT CC
VEGF 90-109 F'	CTT CAT GCC ATC TTG TGT GC
VEGF 226-207 R'	CCA CGA GTT TGT TTC GAT GC
GAPDH 523-542 F'	ACT GTG CAT GCG ATT ACT GC
GAPDH 622-603 R'	AGG CTG GAA TGA TGT TCT GG
AChE 1577-1596 F'	TCA GCA GAA GGA TGA TGC AC
AChE 1708-1689 R'	GAT TGG TGT TGA GTG CGA TG
NP1 209-228 F'	ACA ACA CGA ACC GCT TCA AC
NP1 284-265 R'	TTC TTC TCC TCG CTG TTG TG
CSPE1 1518-1537 F'	TGG CCT GAA TTT AGG AGA CG
CSPE1 1595-1576 R'	TTA ACC CTT TCC GCA CTG AC
HSC70 91-110 F'	AAT GAC CAG GGC AAC AGA AC
HSC70 174-155 R'	CTG ATT TTT AGC GGC GTC TC

**Expression primers**	
EYFP 1-20 + Kpn I F’	GCG CGG TAC CATGGTGAGCAAGGGCGAGGA
EYFP 717-700 + LnkA R’	GCTGCCTCCTGCAGCGGCCGCTCCGGACTTGTACAGCT CGTCCAT
Ps CIRBP 1-23 + LnkA/DP F'	GCT GCA GGA GGC AGC GAT CCC ATG TCT TCA GAT GAT GGG AAG AT
Ps CIRBP 253-274 + LnkA/DP F'	GCT GCA GGA GGC AGC GAT CCC TCT GAC CGA AGC CGA GGC GGC T
Ps CIRBP 252-231 + TAA + Hind R'	CAT GAA GCT TTT ATT TGC CCG CTT GGT CCA CTC GG
Ps CIRBP 498-478 + HindIII R'	GCG CAA GCT TTT AGT TAT CAT AGC TGT CTC T

***in vitro* transcription primers**	
PMF 3' UTR 26-45 + pT7 F'	TAA TAC GAC TCA CTA TAG GTG AGG AAT CAC ACT GGC ATC
PMF 3' UTR 99-118 + pT7 F'	TAA TAC GAC TCA CTA TAG GGG AGC AAA GCT TCT TTG ACG
PMF 3' UTR 271-290 + pT7 F'	TAA TAC GAC TCA CTA TAG GGG GTT AGT GTG ATG GGA AGG
PMF 3' UTR 406-427 + pT7 F'	TAA TAC GAC TCA CTA TAG GTC TGA AAA TGT GGA AGC AGA AA
PMF 3' UTR 288-268 R'	TTC CCA TCA CAC TAA CCC AGT
PMF 3 'UTR 368-349 R'	ACA ACT TGG GTG GCA TCA TT
PMF 3' UTR 565-544 R'	ACA TGG GAA ATT CAG AAA CAG A
PMF 3' UTR 667-647 R'	GCA CCA TCA AAC AAG ACT TCC
Keratin 3' UTR 205-224 + pT7 F’	TAA TAC GAC TCA CTA TAG GGA TTG GCT GAC ATT CCA CCT
Keratin 3' UTR 441-422 R'	CAT TCC CCG GTG TAA GAA TG
